# Enhancing Ankle Movement in Stroke Patients: The Impact of Joint Mobilization Combined with Active Stretching

**DOI:** 10.3390/brainsci15111149

**Published:** 2025-10-27

**Authors:** Shin-Jun Park, Kyun-Hee Cho, Seunghue Oh

**Affiliations:** 1 Department of Physical Therapy, Suwon Women’s University, Hwaseong-si 18333, Republic of Korea; p3178310@swc.ac.kr (S.-J.P.); ckh79@swc.ac.kr (K.-H.C.); 2 Department of Physical Therapy, Uiduk University, Gyeongju-si 38004, Republic of Korea

**Keywords:** stretching exercise, joint mobilization, balance, gait ability, stroke

## Abstract

**Background:** The paralyzed ankles of stroke patients show reduced range of motion, muscle tightness, and joint stiffness, further impeding their ability to maintain balance and walk properly. This study aimed to investigate the effects of a combined joint mobilization and active stretching intervention on ankle stiffness, balance, and gait in patients with stroke. **Methods:** In this study, 24 stroke patients were assigned to two groups of 12 each: the control group (general physical therapy) and the experimental group (joint mobilization technique and active stretching exercise). All interventions were conducted for 30 min a day, 3 times a week, for a total of 4 weeks. Tension and stiffness of the gastrocnemius and tibialis anterior muscles were measured using Myoton^®^PRO. Balance was evaluated using BioRescue, the Berg Balance Scale (BBS), and the Timed-Up and Go tests (TUG). All measurements were evaluated before the start of the intervention and after four weeks. **Results:** The muscle tone and stiffness of the medial and lateral gastrocnemius and tibialis anterior muscles of the experimental group were significantly improved compared with those of the control group. The experimental group had significantly increased moving areas in all directions and BBS scores compared with the control group. The experimental group showed a significant decrease in the time spent on the TUG test compared with the control group. **Conclusions:** We found that joint mobilization combined with active stretching intervention was more effective than general physical therapy in improving ankle joint movement, balance, and gait ability.

## 1. Introduction

Impaired ankle joint movement is the most frequent symptom in stroke patients [1,2,3]. After a stroke, individuals frequently experience deficits in ankle movement, including muscle weakness, spasticity, and altered muscle tone, which directly affect ankle function and mobility [1,3]. Consequently, impaired ankle joint movement leads to a diminished range of motion, muscle tightness, and joint stiffness, further impeding the ability to maintain balance and perform gait [1,4,5].

Specifically, the tibialis anterior (TA) muscle combined with substantial triceps surae spasticity leads to drop foot, which is described as the inability to perform active dorsiflexion during the swing phase of the gait cycle [6,7]. Additionally, spasticity of the gastrocnemius muscle (GCM) is a common post-stroke manifestation characterized by increased muscle tone and involuntary muscle contractions [8]. Gastrocnemius spasticity can lead to stiffness in the calf and ankle joints, causing difficulties in walking and maintaining proper foot positioning [8]. Thus, after a stroke, these impairments can be deficits not only in maintaining balance but also in supporting body weight and facilitating locomotion.

To address these impairments, joint mobilization and stretching interventions have been performed to increase the range of motion for the joint via mechanical changes in the ankle joint, thereby improving balance and gait ability in stroke patients [9,10,11]. Joint mobilization, which creates passive movements on the joint surface, can improve ankle range of motion, balance, and gait function in patients with stroke [12,13]. Stretching exercises are also well documented to improve impaired ankle movements [9,12,14]. In particular, the active stretching method involving active isometric contraction and passive stretching of the agonistic muscle, followed by isometric contraction of the antagonistic muscle, provides better flexibility than passive stretching alone [9,15].

Joint mobilization facilitates posterior talar glide, thereby directly improving ankle dorsiflexion range of motion and improving balance and gait ability [10]. In addition, active stretching induces inhibitory input via Golgi tendon organs and enhances dorsiflexor activation through antagonist isometric contraction [9]. In addition to the Golgi tendon organs, intrafusal muscle fibers (muscle spindles) also play a crucial role in regulating muscle tone and stretch reflexes. While Golgi tendon organs provide inhibitory feedback to prevent excessive muscle contraction, muscle spindles remain active even during relaxation and deliver continuous proprioceptive input to the central nervous system. The combined action of these receptors contributes to muscle tone modulation and improved flexibility during joint mobilization and stretching interventions. These complementary mechanisms are expected to reduce excessive tone and stiffness of the gastrocnemius while reinforcing tibialis anterior activation, ultimately expanding the limit of stability and improving functional balance and gait performance. Based on these mechanisms, we hypothesized that joint mobilization combined with active stretching would lead to greater improvements in ankle range of motion, muscle tone, balance, and gait performance compared to conventional physical therapy in patients with stroke. Even though a few studies have demonstrated joint mobilization combined with stretching [12,16], the effectiveness of joint mobilization combined with active stretching focused on joint stiffness, balance, and gait ability has not been investigated. Therefore, this study aimed to investigate the effects of a combination of joint mobilization and active stretching interventions on ankle joint stiffness, balance, and gait ability in stroke patients.

## 2. Materials and Methods

### 2.1. Participants

The participants were patients admitted to a rehabilitation hospital. To recruit research participants, an announcement was made on the hospital bulletin board. Forty hospitalized patients expressed interest in participating, and 24 of them met the inclusion criteria for this study. The inclusion criteria for selecting study participants were as follows: patients with chronic stroke diagnosed with a stroke within the past 6 months, those who scored ≥24 on the Korean Mini-Mental State Examination, those who had a grade of 1 to 3 on the modified Ashworth scale, and who were able to walk over 10 m. The exclusion criteria for study participants included hospitalization due to stroke recurrence, other neurological diseases such as Parkinson’s disease and cerebellar diseases, orthopedic diseases of the ankle (ankle sprains, fractures, etc.), mental disorders (depression, etc.), and impairments of cognitive executive functions or speech disorders that prevented participants from understanding or following the investigator’s instructions.

### 2.2. Study Procedure

This study was a single blind, randomized controlled trial with two groups. A physical therapist unrelated to the study generated random numbers using a computer. In total, 24 participants were randomly assigned to two groups of 12 each. The patients themselves did not know the treatment group to which they belonged. The control group received general physical therapy, and the experimental group underwent a combination of joint mobilization and active stretching exercises with antagonist isometric contraction. The study period was 30 min a day, three times a week, for a total of four weeks.

In all participants, stiffness and balance variables were evaluated before and 4 weeks after the intervention. All evaluations were performed by a physical therapist who was unrelated to this study and did not know the purpose and hypothesis of the study. The evaluator was blinded to the participants’ group allocation. All study participants provided written informed consent. If the participant had difficulty filling out the consent form owing to arm dysfunction, the guardian had to complete the consent form. This study was approved by the Institutional Review Board of Yongin University (approval no. 2-1040966-AB-N-01-2104-HSR-215-1), Yongin-si, Gyeonggi-do, South Korea.

### 2.3. Intervention

The intervention period for this study was 4 weeks. All participants received all existing treatments, and additional interventions according to the study were performed thrice a week in the afternoon, after all existing treatments were completed. All interventions were performed by a physical therapist with >10 years of clinical experience in physical therapy and >5 years of experience in orthopedic physical therapy. During the evaluation, a physical therapist stood by the patient to prevent falls.

#### 2.3.1. Control Group Intervention Method

In the control group, the intervention was general physical therapy. General physical therapy consisting of trunk exercises, balance training, and gait training was provided to participants for 30 min. General physical therapy was based on neurodevelopmental treatment and included trunk control exercises, balance training using a balance board, and walking forward, backward, and sideways. General physical therapy was conducted based on the physical condition of the study participants.

Trunk control exercises were performed for 10 min while the participant sat on a ball and maintained balance with assistance as needed. Balance training was conducted for 10 min with the participant standing on a balance board and maintaining posture with assistance as needed. Gait training was performed for 10 min by repeatedly walking forward, backward, and sideways along a marked 10 m straight line.

#### 2.3.2. Experimental Group Intervention Method

The experimental group intervention method combined the joint mobilization technique and active stretching exercise with an antagonist muscle isometric contraction exercise. For joint mobilization, the participant was placed in a supine position, keeping the ankle joint in a comfortable state, and fixing the shin part of the paralyzed leg with a fixation belt. The researcher held the heel bone on the paralyzed side with one hand. With the researcher’s opposite hand, the talus bone was grasped on the paretic side by wrapping it around the webbed space from the front. Subsequently, while fixing the foot on the paralyzed side to the researcher’s thigh, a posterior glide of the talus bone with both hands was performed. This condition was maintained for 1 min [5]. At this time, sustained stretching of the talocrural joint was maintained using a grade 3 joint mobilization technique (Kaltenborn joint mobilization). Joint mobilization was rhythmically repeated about 10 times for 15 min.

Active stretching exercises with antagonist muscle isometric contraction were applied to the ankle joint on the paralyzed side. The participant started in the lunge position, holding the mat with the arm extended forward and the heel of the paralyzed side behind. The participant maintained isometric contraction for 6 s with the heel of the paralyzed side touching the floor and then rested for 2 to 3 s. At this time, the physical therapist moved the heel on the paralyzed side further backward according to the stretched muscle condition and performed manual stretching of the ankle joint on the paralyzed side for 15–18 s. Subsequently, the participant maintained isometric contraction of dorsiflexion for 6 s, released the force, returned to the starting position, and rested for 10 s. In this way, the stretching exercise was applied repeatedly about 15 times for 15 min [16,17]. The experimental group received joint mobilization techniques followed by active stretching exercises with isometric contraction of the antagonist muscles. Each intervention was applied for 15 min, resulting in a total intervention duration of 30 min.

### 2.4. Outcome Measures

In this study, muscle tension and stiffness of the medial and lateral GCM and TA muscles were used to measure ankle joint stiffness and stability limit, and the Berg Balance Scale (BBS) and Timed-Up and Go test (TUG) were used. All measurements were evaluated before the start of the intervention and after 4 weeks to analyze the effectiveness of each intervention method.

#### 2.4.1. Muscle Tone and Stiffness Measurement

A portable muscle tension meter (Myoton^®^PRO, MyotonAS, Tallinn, Estonia) was used to measure changes in muscle tone and stiffness of the participants’ medial and lateral GCM and TA muscles. A muscle tension meter was placed vertically on the skin over the muscle, and the response was measured in terms of frequency (Hz), stiffness (N/m), and decay rate [18]. The muscle tension meter is a highly reliable and valid tool for assessing muscle tension and stiffness in stroke patients [19]. However, because the damping rate had a high standard error when measuring [20], the frequency and stiffness were used as the measurement values in this study. Before measuring the muscle tension and stiffness, the participants were allowed to rest for 10 min. The medial and lateral GCM of the study participants were measured in the prone position, whereas the TA muscle was measured in the supine position. The area encompassing the medial and lateral GCM and TA muscles was marked on the measurement area. The measurement site for the medial and lateral GCM was measured after marking the muscle tendon with a marker at one-third of the body between the upper part of the heel bone and the popliteal fold, whereas for the TA muscle, the site was located at the distal one-third of the distance between the lateral malleolus and the lateral prominence. Muscle tendons at one point were marked and measured [21].

#### 2.4.2. Balance Measurement

Balance evaluation (BioRescue, RM Ingenierie, Marseille, France) was used to measure the dynamic balance ability of the study participants. The equipment consisted of a force plate with 1600 pressure sensors and SyCoMoRe software version 8.52. This equipment can measure the movement length (cm), movement area (mm^2^), and stability limit (mm^2^) of the body center in a standing position. The balance measuring instrument is a highly reliable evaluation tool for measuring stability limits, with intraclass correlation coefficients of left (0.78), right (0.76), front (0.69), and rear (0.84) movement areas. However, body center movement length and movement area showed low reliability of <0.6 [22]. Therefore, the stability limit was measured and used as the result in this study. To measure the dynamic balance ability, the participants were asked to stare at a monitor while standing on a force plate. The stability limits were measured by moving the body weight in eight directions: left, right, front, back, and diagonal, as indicated on the monitor. In addition, the left and right sides were measured separately as the paralyzed side and the non-paretic side. If the participant’s foot fell off the footrest during the measurement, the measurement was repeated. All data were collected three times, and the average value was used as the measurement value.

#### 2.4.3. BBS

BBS was used to measure the static and dynamic balance abilities of the study participants. BBS is a highly reliable evaluation tool for balance evaluation in stroke patients, with an intraclass correlation coefficient of 0.98 during test–retest [23]. BBS, which measures the static and dynamic balance abilities of research participants, consists of 14 items divided into three areas: sitting, standing, and posture change. Each detailed item for measuring static balance ability consisted of sitting on one’s own without leaning on the back of a chair, standing without assistance, standing with the eyes closed, and standing with both feet together. The detailed items for measuring dynamic balance ability were standing from sitting, sitting from standing, moving from chair to chair, straightening and extending arms in a standing position, lifting an object from the floor in a standing position, and turning left and right in a standing position. This consists of viewing, rotating 360° from a standing position, alternately placing both feet on a footrest in a standing position, standing without support with one foot in front of the other, and standing on one foot. The score for each item ranges from 0 to a maximum of 4, with a total score of 56; higher scores indicate better balance ability [24].

#### 2.4.4. Timed up and Go Test

The TUG test was used to measure the functional balance ability of the study participants. The TUG test is a highly reliable evaluation tool with an intraclass correlation coefficient of 0.98 during test–retest for stroke patients [25]. To perform the get-up-and-walk test, the research participant sat on a chair without armrests and assumed a starting position with the hip and knee joints flexed at 90°. When the evaluator gave the command “start”, the research participant got up from the chair, walked along a 3 m walking path, returned to the turning point, and sat down on the chair [26]. The evaluator recorded the start time and time to sit back down on the chair in seconds using a stopwatch (HJ, Stopwatch, Guangdong, China). Two measurements were performed, and the average value was used as the measurement value.

### 2.5. Data and Statistical Analysis

The results of this study were analyzed using the SPSS 21.0 program for Windows (IBM^®^, SPSS Statistics, New York, NY, USA). Among the analyses of the general characteristics of the study participants, the chi-square test was used to test for homogeneity for gender, paretic side, and stroke type, vascular damage area, and variables such as age, height, weight, stroke period, and the Korean version of the Mini-Mental State Examination (MAS) were analyzed using descriptive statistics. The results of the Kolmogorov–Smirnov test showed that all variables followed a normal distribution. An independent *t*-test was used to compare differences in variables between the study participants’ intervention methods. A paired *t*-test was used to compare the differences between the study participants before and after each intervention. Statistical significance level was set at *p* < 0.05. All data were analyzed based on the intention to treat (ITT) principle, including all participants initially randomized, regardless of whether they completed the intervention according to the protocol.

## 3. Results

A summary of the demographic and clinical characteristics of stroke patients is presented in Table 1. The changes in muscle tone and stiffness between the groups are shown in Table 2. In the experimental group, the muscle tone and stiffness values in the GCM on the paretic side showed a significant decrease post-intervention (*p* < 0.05). The tone and stiffness values of the TA muscle were significantly higher post-intervention (*p* < 0.05). In comparison, the tone and stiffness of the medial and lateral GCM were significantly decreased compared to those of the control group (*p* < 0.05), and the tone and stiffness of the TA muscle were significantly increased compared to those of the control group (*p* < 0.05).

The balance measurements between groups are shown in Table 3. The movement areas on the paretic, non-paretic, front, and back sides significantly increased after the intervention (*p* < 0.05). The experimental group showed significantly greater movement areas in all directions and higher BBS scores compared with the control group (*p* < 0.05).

The BBS scores are shown in Table 4. The BBS score increased significantly after the intervention (*p* < 0.05). The experimental group had a significantly higher BBS score than the control group (*p* < 0.05).

In the TUG test (Table 5), the time spent after the intervention significantly decreased in both groups (*p* < 0.05). The experimental group spent significantly less time compared to the control group (*p* < 0.05).

## 4. Discussion

In the current study, we investigated joint mobilization combined with active stretching interventions in stroke patients. We found that joint mobilization combined with active stretching intervention for four weeks was more effective than general physical therapy in improving ankle joint movement, balance, and gait ability.

Specifically, the combination of joint mobilization and active stretching resulted in a significant decrease in the tone and stiffness of the GCM on the paretic side after the intervention, indicating improved muscle flexibility [5,9,13,16,27]. Conversely, there was a significant increase in the tone and stiffness of the TA muscle post-intervention, which may suggest enhanced muscle activation and improved motor control by isometric contraction [1,13,28]. According to previous studies, isometric contraction applied immediately before stretching enhances muscle flexibility more effectively than static stretching by activating the Golgi tendon organs and reducing muscle activity. Additionally, the isometric contraction of antagonistic muscles reportedly boosts muscle strength [29]. For instance, enhanced strength of the anterior tibial muscle can improve gait performance in the TUG test [13,30]. These results are consistent with our findings, demonstrating that the active stretching intervention used in this study effectively modulated muscle tone and stiffness, promoted functional movement patterns in stroke patients, reduced gastrocnemius tension, and enhanced tibialis anterior activation.

Joint mobilization facilitates the expansion of the joint cavity by stretching non-contractile tissues, potentially enhancing the range of motion in the ankle joints of stroke patients. Many previous studies have demonstrated that joint mobilization has been shown to improve ankle joint flexibility, which is consistent with the findings of the current study [10,14,15].

The increase in movement area on both the paretic and non-paretic sides, as well as improvements in balance performance measured using the BBS, signify enhanced functional mobility and postural stability following the intervention [13]. Furthermore, significant reductions in the time spent on the TUG test reflect enhanced gait efficiency and speed, indicating improved gait ability and functional capacity [26]. A previous study demonstrated that joint mobilization improved sit-to-stand performance in terms of speed [31]. Another previous study showed that a GCM stretching intervention with ankle dorsiflexion exercises can help decrease the time of the TUG test [32]. It is believed that the combined intervention method caused an overlap of the mechanical and neurophysiological effects through joint mobilization and extension exercises, thereby increasing the range of dorsiflexion more than that in the control group. These improvements are crucial for stroke patients to regain independence in activities of daily living and reduce the risk of falls, which are common complications post-stroke. Although the MyotonPRO and BioRescue systems used in this study are clinically reliable and practical tools for assessing muscle tone and balance, they have limitations in measuring detailed biomechanical parameters. Therefore, future studies should employ more advanced motion analysis systems to provide a deeper understanding of the mechanisms underlying improvements in motor function after rehabilitation interventions.

The observed improvements in gait and balance may be attributed to several physiological mechanisms. Joint mobilization facilitates posterior talar glide and capsular extensibility, restoring normal arthrokinematics and promoting ankle dorsiflexion, which enhances postural stability and step initiation [33,34]. In addition, active stretching induces inhibitory effects on alpha motor neurons through Golgi tendon organ activation while enhancing proprioceptive feedback from muscle spindles, leading to reduced hypertonicity and improved coordination between the tibialis anterior and gastrocnemius muscles [35,36]. These complementary mechanisms may expand the limits of stability and improve functional balance and gait performance in stroke patients [37,38].

This study has several limitations. The number of participants was relatively small (n = 24), which may have reduced the statistical power to detect small effects and limited the generalizability of the results. However, as a preliminary clinical investigation, the sample size was determined based on feasibility and previous studies with similar intervention protocols. According to established recommendations for pilot or exploratory trials [39], a sample size of approximately 10–12 participants per group is acceptable when the expected standardized effect size is large (≥0.8). The observed effect sizes in this study exceeded this threshold, suggesting sufficient power to detect large intervention effects. Additionally, the intervention period was relatively short (four weeks), and no follow-up assessments were conducted to evaluate the long-term maintenance of participants’ improvements. Therefore, the long-term effects of the intervention could not be determined. Finally, a potential placebo effect cannot be excluded, as participants’ motivation and engagement levels may have varied across individuals.

## 5. Conclusions

The present study demonstrated that a four-week intervention combining joint mobilization with active stretching was more effective than general physical therapy in improving ankle muscle tone, stiffness, balance, and gait ability in stroke patients. These results suggest that the integrated approach effectively modulates both mechanical and neurophysiological components of motor control, contributing to enhanced functional mobility. The findings highlight the clinical applicability of combining joint mobilization and active stretching as a feasible and efficient intervention strategy for stroke rehabilitation. Future studies with larger sample sizes and long-term follow-up assessments are warranted to confirm these effects and explore the underlying mechanisms in greater detail.

## Figures and Tables

**Table 1:** General characteristics of subjects.

Classification	Control Group	Experimental Group	*p*
Gender (male/female)	7/5	8/4	1.000 ^a^
Paretic side (left/right)	4/8	4/8	1.000 ^a^
Type (hemorrhage/infarction)	4/8	2/10	0.640 ^a^
Vascular damage area (ACA/MCA/PCA/Brain Stem/Basal Ganglia)	1/5/1/2/3	1/4/2/1/4	0.922 ^a^
Age (years)	62.67 ± 7.96	63.75 ± 7.39	0.733 ^b^
Height (cm)	164.92 ± 9.63	164.25 ± 7.68	0.853 ^b^
Weight (kg)	69.42 ± 8.11	69.58 ± 6.92	0.957 ^b^
Onset period (month)	12.58 ± 2.68	12.42 ± 2.78	0.882 ^b^
K-MMSE (score)	26.50 ± 1.24	26.92 ± 1.31	0.433 ^b^
MAS (score)	2.00 ± 0.85	1.92 ± 0.90	0.818 ^b^

All values are presented as mean and standard deviation. ^a^ Chi-square test between two intervention groups. ^b^ Independent *t*-test between the two intervention groups. ACA: Anterior cerebral artery. MCA: Middle cerebral artery. PCA: Posterior cerebral artery. K-MMSE: Korean mini-mental state examination. MAS: Modified Ashworth scale.

**Table 2:** Comparison of muscle tone, stiffness pre-test and post-test between groups.

Classification		Pre-Test	Post-Test	Change	95% CI ^b^	Effect Size
MGCM Muscle Tone (Hz)						
PS	Control group	17.18 ± 2.52	17.18 ± 3.03	−0.01 ± 0.72	1.13 (0.30, 1.95)	1.17
	Experimental group	16.42 ± 1.96	15.28 ± 1.62	−1.13 ± 1.15 * ^a^		
N-PS	Control group	15.69 ± 2.53 ^‡^	15.58 ± 2.69 ^‡^	−0.12 ± 0.81	0.092 (−0.64, 0.82)	0.11
	Experimental group	14.75 ± 1.54 ^‡^	14.54 ± 1.00	−0.21 ± 0.90		
LGCM Muscle Tone (Hz)						
PS	Control group	17.98 ± 2.06	17.96 ± 2.32	−0.03 ± 0.81	1.33 (0.27, 2.40)	1.08
	Experimental group	17.28 ± 2.19	15.92 ± 2.45	−1.36 ± 1.54 * ^a^		
N-PS	Control group	16.28 ± 1.92 ^‡^	16.27 ± 2.14 ^‡^	−0.02 ± 0.82	0.33 (−0.45, 1.11)	0.36
	Experimental group	15.94 ± 1.48 ^‡^	15.59 ± 1.40	−0.35 ± 1.01		
TA Muscle Tone (Hz)						
PS	Control group	18.03 ± 2.42	18.03 ± 2.44	0.01 ± 0.61	−0.92 (−1.68, −0.15)	1.02
	Experimental group	18.07 ± 2.50	18.99 ± 2.36	0.93 ± 1.11 * ^a^		
N-PS	Control group	20.34 ± 1.37 ^‡^	20.58 ± 1.29 ^‡^	0.24 ± 1.21	−0.26 (−1.16, 0.65)	0.24
	Experimental group	20.03 ± 2.32 ^‡^	20.53 ± 2.32	0.50 ± 0.89		
MGCM Stiffness (N/m)						
PS	Control group	317.75 ± 62.73	316.58 ± 63.15	−1.17 ± 9.18	30.92 (7.12, 54.72)	1.10
	Experimental group	297.58 ± 52.05	265.50 ± 30.63	−32.08 ± 38.68 * ^a^		
N-PS	Control group	284.00 ± 50.21 ^‡^	287.75 ± 54.54 ^‡^	3.75 ± 16.59	−0.58 (−13.72, 12.56)	0.04
	Experimental group	256.42 ± 24.24 ^‡^	260.75 ± 17.36	4.33 ± 14.33		
LGCM Stiffness (N/m)						
PS	Control group	328.00 ± 58.10	325.08 ± 47.22	−2.92 ± 24.97	31.42 (8.01, 54.82)	1.14
	Experimental group	315.92 ± 53.95	281.58 ± 52.78	−34.33 ± 29.97 * ^a^		
N-PS	Control group	306.00 ± 43.55 ^‡^	302.17 ± 43.44 ^‡^	−3.83 ± 17.95	5.17 (−10.88, 21.22)	0.27
	Experimental group	287.33 ± 27.98 ^‡^	278.33 ± 20.80	−9.00 ± 19.89		
TA Stiffness (N/m)						
PS	Control group	318.50 ± 54.46	321.25 ± 54.66	2.75 ± 10.47	−25.83 (−46.82, −4.85)	1.08
	Experimental group	322.50 ± 55.25	351.08 ± 52.34	28.58 ± 32.06 * ^a^		
N-PS	Control group	378.58 ± 40.42 ^‡^	386.33 ± 47.09 ^‡^	7.75 ± 36.35	4.08 (−29.73, 37.90)	0.01
	Experimental group	391.25 ± 38.32 ^‡^	394.92 ± 49.85	3.67 ± 43.11		

All values are presented as mean and standard deviation. ^‡^ Significant difference between paretic side and non-paretic side. * Significant difference between pre-test and post-test (*p* < 0.05). ^a^ Significant difference between control group and experimental group (*p* < 0.05). ^b^ 95% confidence interval. MGCM: Medial gastrocnemius, LGCM: Lateral gastrocnemius, TA: Tibialis anterior. PS: Paretic side, N-PS: Non-paretic side.

**Table 3:** Comparison of limited of stability pre- and post-test in two groups.

Classification	Pre-Test	Post-Test	Change	95% CI ^b^	Effect Size
Paretic Side Area (mm^2^)					
Control group	680.75 ± 139.20	1118.33 ± 162.85	437.58 ± 104.80 *	−222.00 (−362.52, −81.48)	1.34
Experimental group	660.75 ± 173.68	1320.33 ± 305.72	659.58 ± 210.01 * ^a^		
Non-Paretic Side Area (mm^2^)					
Control group	1300.75 ± 192.20	1629.17 ± 267.63	328.42 ± 182.68 *	−413.83 (−590.14, −237.53)	1.99
Experimental group	1258.67 ± 184.20	2000.92 ± 262.74	742.25 ± 230.98 * ^a^		
Forward Area (mm^2^)					
Control group	1220.67 ± 147.19	1609.00 ± 248.71	388.33 ± 182.29 *	−328.92 (−487.28, −170.56)	1.76
Experimental group	1198.92 ± 168.34	1916.17 ± 269.76	717.25 ± 191.67 * ^a^		
Backward Area (mm^2^)					
Control group	760.83 ± 186.28	1121.83 ± 243.30	361.00 ± 189.42 *	−323.58 (>−548.70, −98.47)	1.22
Experimental group	720.50 ± 230.65	1405.08 ± 356.13	684.58 ± 324.82 * ^a^		
Total Area (mm^2^)					
Control group	1981.50 ± 311.24	2747.50 ± 399.24	766.00 ± 241.24 *	−635.83 (−922.57, −349.10)	1.88
Experimental group	1919.42 ± 301.41	3321.25 ± 520.94	1401.83 ± 413.76 * ^a^		

All values are presented as mean and standard deviation. * Significant difference between pre-test and post-test (*p* < 0.05). ^a^ Significant difference between control group and experimental group (*p* < 0.05). ^b^ 95% confidence interval.

**Table 4:** Comparison of BBS pre- and post-test in two groups.

Classification	Pre-Test	Post-Test	Change	95% CI ^b^	Effect Size
BBS (Score)					
Control group	35.17 ± 3.49	38.08 ± 3.42	2.92 ± 1.78 *	−3.08 (−5.36, −0.81)	1.15
Experimental group	36.58 ± 4.21	42.58 ± 2.43	6.00 ± 3.36 * ^a^		

All values are presented as mean and standard deviation. * Significant difference between pre-test and post-test (*p* < 0.05). ^a^ Significant difference between control group and experimental group (*p* < 0.05). BBS: Berg balance scale test. ^b^ 95% confidence interval.

**Table 5:** Comparison of TUG pre- and post-test in two groups.

Classification	Pre-Test	Post-Test	Change	95% CL ^b^	Effect Size
TUG (s)					
Control group	30.75 ± 4.07	27.17 ± 4.47	−3.58 ± 3.58 *	6.00 (2.58, 9.42)	1.49
Experimental group	29.75 ± 4.79	20.17 ± 3.43	−9.58 ± 4.46 * ^a^		

All values are presented as mean and standard deviation. * Significant difference between pre-test and post-test (*p* < 0.05). ^a^ Significant difference between control group and experimental group (*p* < 0.05). TUG: Timed up and go test. ^b^ 95% confidence interval.

## Data Availability

The data presented in this study are available upon request from the corresponding author due to privacy and ethical restrictions.
